# Histopathological Injuries, Ultrastructural Changes, and Depressed TLR Expression in the Small Intestine of Broiler Chickens with Aflatoxin B_1_

**DOI:** 10.3390/toxins10040131

**Published:** 2018-03-21

**Authors:** Fengyuan Wang, Zhicai Zuo, Kejie Chen, Caixia Gao, Zhuangzhi Yang, Song Zhao, Jianzhen Li, Hetao Song, Xi Peng, Jing Fang, Hengmin Cui, Ping Ouyang, Yi Zhou, Gang Shu, Bo Jing

**Affiliations:** 1 College of Veterinary Medicine, Sichuan Agricultural University, Chengdu 611130, China; wfy_sccd@163.com (F.W.); zzcjl@126.com (Z.Z.); m15009661712@163.com (C.G.); bingozhaosong@163.com (S.Z.); sht854844223@sina.com (H.S.); cuihengmin2008@sina.com (H.C.); ouyang.ping@163.com (P.O.); dyysg2005@sicau.edu.cn (G.S.); jingbo@sicau.edu.cn (B.J.); 2 School of Public Health, Chengdu Medical College, Chengdu 610500, China; ckj930@126.com; 3 Animal Research Institute, Chengdu Academy of Agriculture and Forestry Sciences, Chengdu 611130, China; yangzhuangzhi8@163.com; 4 Department of Preventive Veterinary, Chengdu Agricultural College, Chengdu 611130, China; jianzhenli2006@163.com; 5 College of Life Sciences, China West Normal University, Nanchong 637002, China; 6 Life Science Department, Sichuan Agricultural University, Yaan 625014, China; 13981616210@139.com

**Keywords:** aflatoxin B_1_, small intestine, histopathological lesions, ultrastructural changes, toll-like receptors

## Abstract

To explore AFB_1_-induced damage of the small intestine, the changes in structure and expression of TLRs (Toll-like Receptors) in the small intestine of chickens were systematically investigated. Ninety healthy neonatal Cobb chickens were randomized into a control group (0 mg/kg AFB_1_) and an AFB_1_ group (0.6 mg/kg AFB_1_). The crypt depth of the small intestine in the AFB_1_ group was significantly increased in comparison to the control chickens, while the villus height and area were evidently decreased, as well as the villus:crypt ratio and epithelial thickness. The histopathological observations showed that the villi of the small intestine exposed to AFB_1_ were obviously shedding. Based on ultrastructural observation, the absorptive cells of small intestine in the AFB_1_ group exhibited fewer microvilli, mitochondrial vacuolation and the disappearance of mitochondrial cristae, and junctional complexes as well as terminal web. Moreover, the number of goblet cells in the small intestine in the AFB_1_ group significantly decreased. Also, AFB_1_ evidently decreased the mRNA expression of TLR2-2, TLR4, and TLR7 in the small intestine. Taken together, our study indicated that dietary 0.6 mg/kg AFB_1_ could induce histopathological injuries and ultrastructural changes, and depress levels of TLR mRNA in the chicken small intestine.

## 1. Introduction

Aflatoxins, secondary metabolites produced by some *Aspergillus* species [1,2], are important mycotoxins since about 0.5 to 4.5 billion people are exposed to high levels of aflatoxins. [3,4]. Aflatoxin B_1_ (AFB_1_) is the main source and most toxic type of aflatoxins [5]. The liver is the main target organ for aflatoxins [6,7]. The negative effects of AFB_1_ have also been well-documented, including reduced performance, decreased immune system function, and increased susceptibility to diseases in several animal species [8,9,10].

Conversion and absorption of food components mainly take place in the gastrointestinal tract. Normal nutrient supply is supported by several factors, including absorptive surfaces, residing microorganisms, and host-derived physiological processes [10]. Meanwhile, the intestinal mucosa is continually exposed to a series of antigens, especially the bacterial antigens. Intestinal epithelial cells (IECs) act as a defense system between the intestinal lumen and the lamina propria [11]. The entire structure and function of IECs, which possess tight junction and goblet cells producing mucus, prevent luminal antigens from translocating to the subepithelial tissue [12,13].

The immunotoxicity of AFB_1_ to the intestine has drawn research attention. It has been reported that AFB_1_ decreased the proportion of T-cell subset and number of IgA^+^ cells in the small intestine of chickens [14,15]. The literature, however, is scanty and controversial as to the effects of AFB_1_ on the morphology and histopathology of the gastrointestinal tract [10]. After exposure to 0.02 mg/kg or 0.7 mg/kg AFB_1_ for three weeks, the density (weight/length) of the whole intestine in chickens was evidently decreased [16,17]. Grozeva et al. found that 0.5 mg/kg AFB_1_ induced generalized hyperaemia and mononuclear cell infiltration in the small intestine of broilers within the 42 days of an experiment [18], but, at a higher level of 4 mg/kg diet, Ledoux et al. revealed no histological damage to male broilers’ small intestine after a three-week exposure [19]. 

The pattern recognition receptors (PRRs) have been confirmed to be associated with immunity, inflammation, and cancer [20]. TLRs, one type of the PRRs, have been studied in different areas, including immunotoxicity, inflammation, oxidative stress and cell survival [21]. A few in vitro studies reported that mycotoxins affected TLRs’ expression or TLRs-associated pathways. Mixed aflatoxins B and G up-regulated TLR2 and TLR4 transcript in human peripheral blood mononuclear cells [21], while deoxynivalenol led to the inhibition of TLR-MyD88 signaling in RAW264 cells [22]. 

The gastrointestinal tract is the first organ by which AFB_1_ comes into the body of human and animals; thus, compared with other organs, this toxin should exert greater impacts on the small intestine. However, the effects of AFB_1_ on the small intestine are often neglected and inconclusive [10]. And the doses and exposure time of AFB_1_ to cause histopathological changes of the intestine were controversial in chickens [16,17,18,19]. Our team’s research has shown that 0.15 mg/kg, 0.3 mg/kg and 0.6 mg/kg AFB_1_ could cause obvious toxic effects on chichen immune organs with dose-response [9,23,24]. In our previous study, 0.3 mg/kg AFB_1_ decreased jejunal villus height, villus height/crypt ratio, and induced shedding of epithelial cells on the tip of chicken jejunal villus from 7 to 21 days of age [25]. To observe whether 0.6 mg/kg AFB_1_ would cause more serious damage to the whole small intestine in the same exposure time, we determined to use a higher dose (0.6 mg/kg AFB_1_) to do the further systemic research. Furthermore, TLRs play vital roles in the innate immune system, and several studies on the effects of different mycotoxins on TLR gene expression were focused on TLR2, TLR4 and TLR7 [21,22,26,27,28]. Thus, these three TLRs were chosen for this research in order to compare the AFB_1_-induced effects on TLR2, TLR4 and TLR7 with other mycotoxins.

Therefore, this research was conducted to systematically study the histopathological damages and TLR expression in the small intestine of chickens caused by dietary 0.6 mg/kg AFB_1_ through multiple technologies, such as hematoxylin and eosin staining, histological chemistry, microscopic analyses of the villus, crypt depth and goblet cells, the mucosal epithelium observation by transmission electron microscope, and TLR2, TLR4, and TLR7 mRNA expression by qRT-PCR.

## 2. Results

### 2.1. Morphological Measurements in the Small Intestine

**Figure 1:**
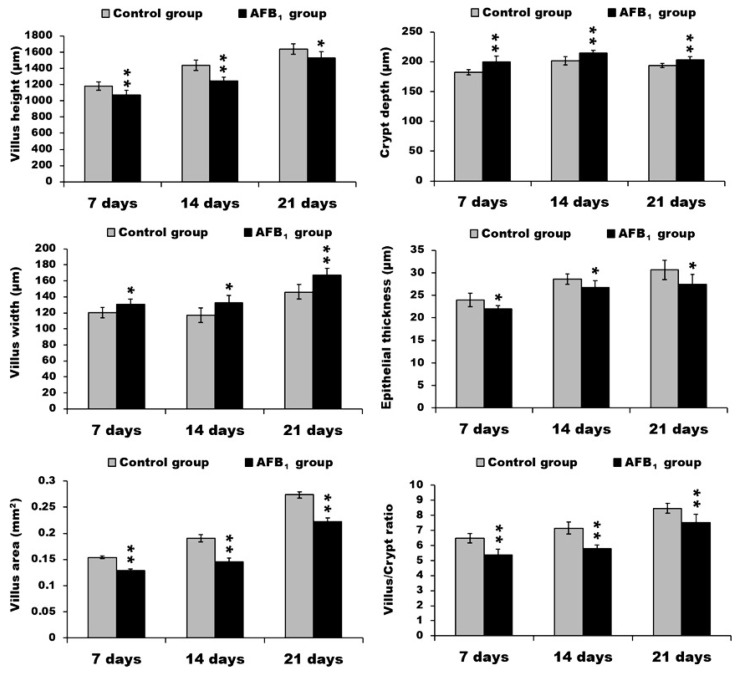
The data of villus height, crypt depth, villus width, epithelial thickness, villus area, and villus/crypt ratio in the duodenum. * *p* < 0.05, ** *p* < 0.01.

At three different time points, compared with the control group, the villus height of duodenum in the AFB_1_ group was significantly decreased (*p* < 0.05 or *p* < 0.01), while the villus width was significantly increased (*p* < 0.05 or *p* < 0.01). Overall, the villus area of the duodenum in the AFB_1_ group was evidently decreased (*p* < 0.01). Although the crypt depth of duodenum in the AFB_1_ group was significantly increased (*p* < 0.01), the villus:crypt ratio in the AFB_1_ group dropped significantly (*p* < 0.01). Moreover, the epithelial thickness of duodenum in the AFB_1_ group significantly declined (*p* < 0.05) (Figure 1).

**Figure 2:**
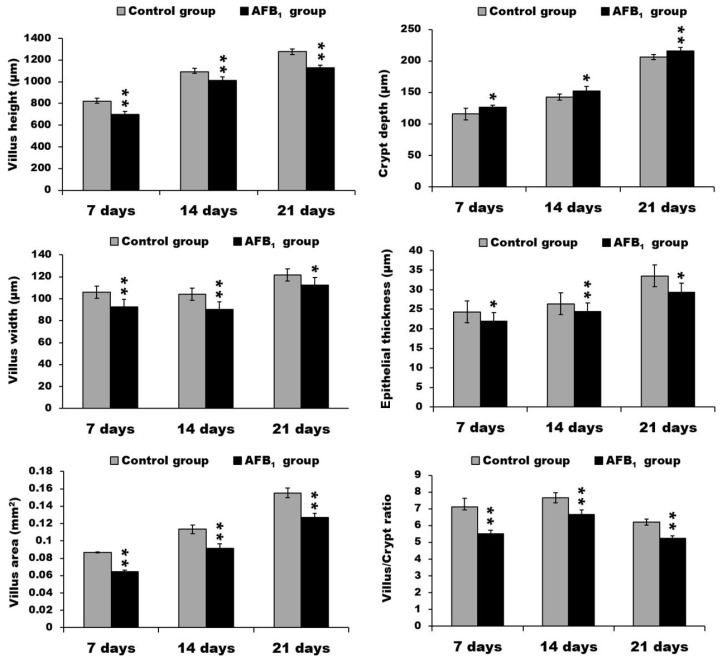
The data of villus height, crypt depth, villus width, epithelial thickness, villus area, and villus/crypt ratio in the jejunum. * *p* < 0.05, ** *p* < 0.01.

Compared with the control group, the jejunum of the AFB_1_ group exhibited lower villus height, width, and area (*p* < 0.05 or *p* < 0.01). The jejunal crypt depth in the AFB_1_ group, moreover, was significantly increased (*p* < 0.05 or *p* < 0.01), in addition, the villus:crypt ratio was evidently lower (*p* < 0.01). At all three time points, the epithelial thickness of jejunum in the AFB_1_ group was significantly decreased (*p* < 0.05 or *p* < 0.01) (Figure 2). 

**Figure 3:**
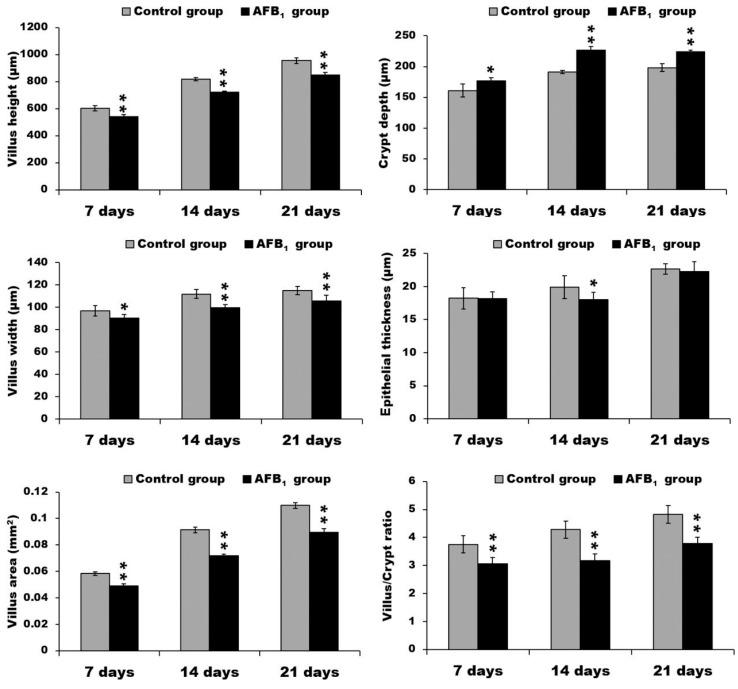
The data of villus height, crypt depth, villus width, epithelial thickness, villus area, and villus/crypt ratio in the ileum. * *p* < 0.05, ** *p* < 0.01.

Compared with the control group, the ileum of broilers exposed to AFB_1_ showed lower villus height, width, and area (*p* < 0.05 or *p* < 0.01). The ileac crypt depth in the AFB_1_ group, moreover, was significantly increased (*p* < 0.05 or *p* < 0.01), and the villus:crypt ratio had evidently dropped (*p* < 0.01). On day 14, the epithelial thickness of the ileum in the AFB_1_ group was significantly decreased (*p* < 0.05) (Figure 3). 

### 2.2. Histopathological Analysis

**Figure 4:**
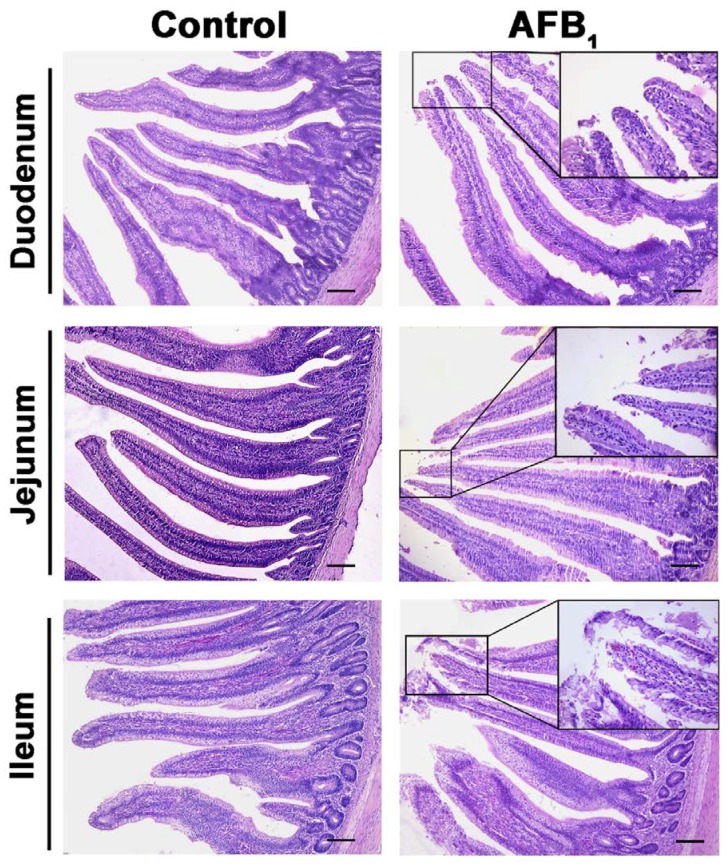
The representative microstructure of intestinal villi at 21 days of age. Note: The enlarged box shows the shedding of epithelial cells; H.E. stain, scale bar = 200 μm.

The apical epithelia of villi in the small intestine in the AFB_1_ group were shedding (Figure 4). No other obvious pathological damage to the chicken intestine in the AFB_1_ group was observed.

### 2.3. Ultrastructure Changes

**Figure 5:**
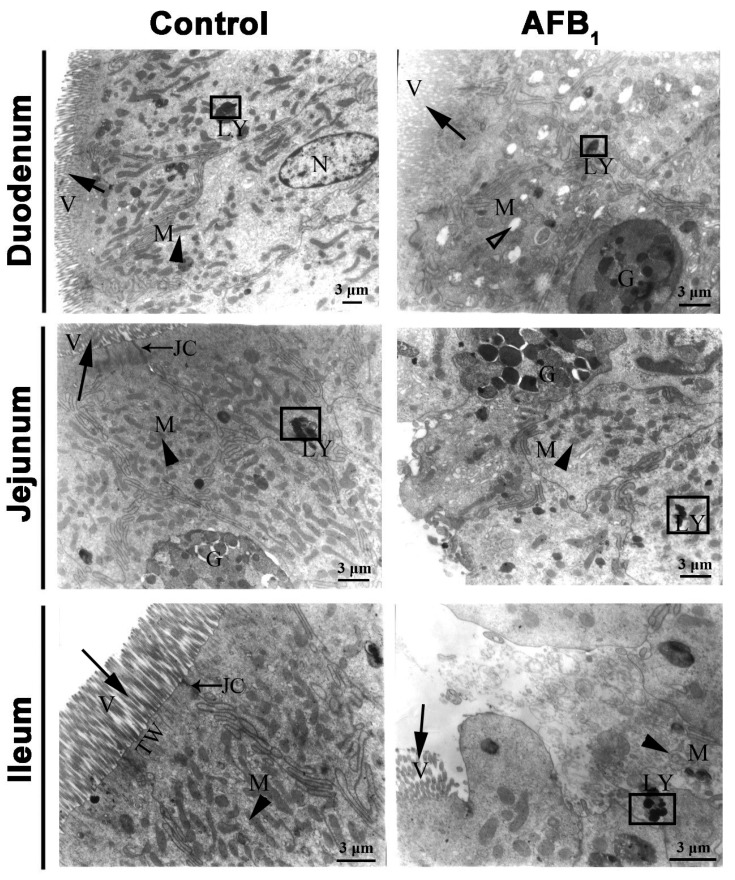
The representative ultrastructure of absorptive cells and goblet cells in the small intestine at 21 days of age. Note: M: mitochondria (▲), V: microvilli (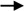), G: Goblet cells, LY: lysosomes (□), N: nucleus, JC: Junctional complexes (→), TW: terminal web. Scale bar = 3 μm.

The mucosal epithelium of the small intestine consists predominately of absorptive cells and goblet cells (Figure 5). In the duodenum, jejunum, and ileum of the control group on day 21 of the experiment, there are closely packed microvilli in the apical border of absorptive cell. Also, abundant mitochondria with normal ultra-structure were located in the apical cytoplasm of this cell. Junctional complexes including tight junction, intermediate junction, and desmosome were distributed between the epithelial cells at the luminal surface. A terminal web containing many micro-filaments was well organized under the microvilli. 

In the duodenum of the AFB_1_ group on day 21, reduced mitochondrial cristae and mitochondrial vacuolation and lysis of mitochondrial contents in the apical portion of some absorptive cells were the most obvious ultrastructural pathological changes (Figure 5).

In the jejunum of the AFB_1_ group, the microvilli on the surface of some absorptive cells were completely shed, and the junctional complexes and terminal web were partly or completely disappeared. In addition, the number of mitochondria had evidently decreased (Figure 5).

Also, the microvilli of some ileac absorptive cells in the AFB_1_ group on day 21 were fewer or shorter, or even completely shed, and the junctional complexes and terminal web had partially or entirely disappeared. Finally, fewer mitochondria, a decreased electron density, and lysis contents of apical cytoplasm were observed (Figure 5).

### 2.4. Number of Goblet Cells Shown by Alcian Blue/PAS

**Figure 6:**
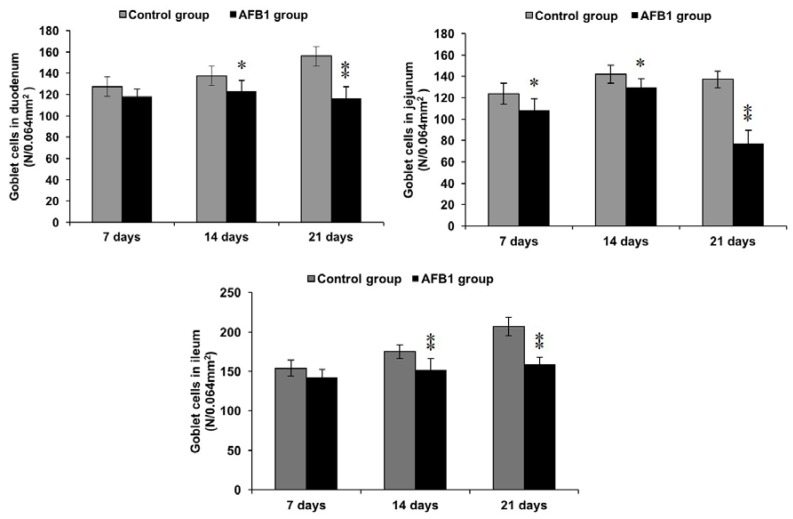
The numbers of goblet cells in the small intestine. Note: 0.064 mm^2^ was the area of one field under 400× magnification. * *p* < 0.05, ** *p* < 0.01.

**Figure 7:**
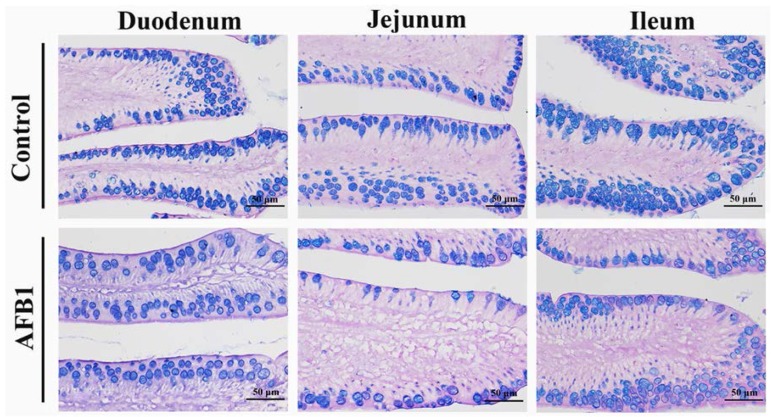
The representative goblet cells in the mucous epithelial cells of villi in the small intestine on day 21 (Alcian Blue/PAS stain, scale bar = 50 μm).

By Alcian Blue/PAS stain, goblet cells locating in mucosal epithelia and crypt were blue or purple in the small intestine of both groups. Compared with the control group, on day 7, the number of goblet cells in jejunum of the AFB_1_ group was significantly decreased (*p* < 0.05). On days 14 and 21, the numbers of goblet cells in the AFB_1_ group were evidently decreased (*p* < 0.05 or *p* < 0.01) (Figure 6 and Figure 7). 

### 2.5. mRNA Expression of TLR2-2, TLR-4 and TLR-7 

**Figure 8:**
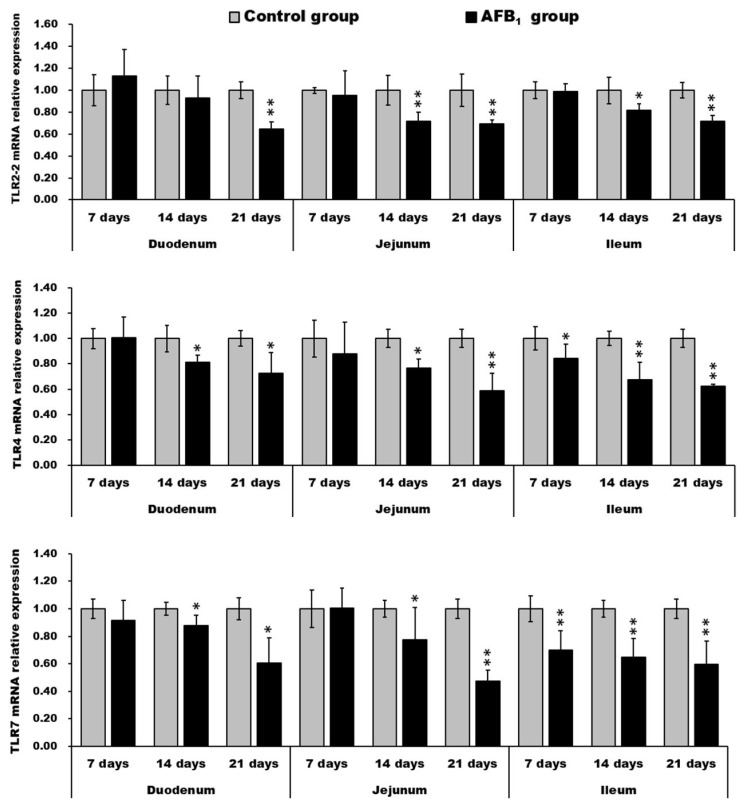
mRNA expression levels (fold of the control) of the TLR2-2, TLR4, and TLR7 in the small intestine. * *p* < 0.05, ** *p* < 0.01.

The mRNA expression of TLR2-2, TLR-4, and TLR7 in both the duodenum and jejunum in the AFB_1_ group significantly decreased in comparison to the control group on day 14 and 21 (*p* < 0.05 or *p* < 0.01), except for duodenal TLR2-2 mRNA expression on day 14. The value of ileac TLR2-2 was evidently decreased in the AFB_1_ group on days 14 and 21 (*p* < 0.05 or *p* < 0.01), and the values of ileac TLR-4 and TLR-7 in the AFB_1_ group significantly declined during the experiment (*p* < 0.05 or *p* < 0.01) (Figure 8).

## 3. Discussion

Aflatoxin B_1_, the most common aflatoxin, commonly contaminates various kinds of human food and animal feed elements in tropical and subtropical areas [10]. AFB_1_ can enter into animals and humans by consumption of AFB_1_-contaminated feed or food, so the gastrointestinal is the first site to contact AFB_1_, especially the small intestine [18]. The mucosal layer of the small intestine, including the lining epithelium, lamina propria with gland, and lamina muscularis, has the specificity to be structured in a way that provides a large surface, thus maximizing the absorption of nutrients. The surface of the mucosa is studded with finger-like projections, the intestinal villi, which are the most characteristic feature of the small intestine [29]. The crypts opening between the bases of the villi penetrate the mucosa as far as the lamina muscularis [29]. The intestinal villus and crypt play a crucial role in nutritional absorption and animal growth [30]. The continuous regeneration of the small intestinal epithelium is ensured by the migration of proliferating crypt cells up the villi [31]. Therefore, the height, width, and area of villus, but especially the area, are positively related with the absorptive efficiency of the small intestine in chickens, as well as epithelium thickness and villus:crypt ratio, while the crypt depth is negatively related. Our study showed decreased villus height and area as well as villus:crypt ratio in the three parts of the small intestine in the AFB_1_ group in comparison with the control chickens, suggesting that AFB_1_ reduced the small intestine’s surface area for absorption in chickens. Our morphological measurements were similar to most previous studies, e.g., one in which Zhang et al. found that 0.3 mg/kg AFB_1_ could induce slightly decreased jejunal villus height and shedding of epithelial cells on the tip of the jejunal villus [25]; Aboutalebi reported that 0.7 mg/kg AFB_1_-treatment induced more serious damage in the duodenum than 0.35 mg/kg AFB_1_-treatment [32]. Thus, AFB_1_ may have a dose-dependent effect. However, Feng et al. reported that AFB_1_ could increase the villus height and area in the duodenum and jejunum [33], which may result from differences in animal species and doses of AFB_1_ used for experiments.

In this study, moreover, AFB_1_ caused the shedding of the apical epithelia of villi in the small intestine, which was also observed by other researchers in chickens [18,25]. In murine models, AFB_1_ has been found to lead to pathological damage of the intestinal mucosa [34], and to decreased cell proliferation [35]. Furthermore, Akinrinmade et al. observed leucocyte, lymphocyte, and mononuclear cell infiltration in the lamina propria of rats when AFB_1_ was administered intraperitoneally [36], which was not observed in our study. These discrepancies may be attributed to different methods of administration and different animal models. 

In addition, to explore the relationship between AFB_1_ and damage to absorptive cells in the small intestine, we used a transmission electron microscope to examine the ultrastructure of the epithelial cells of the small intestine following AFB_1_ exposure. In the duodenum, fewer mitochondrial cristae of absorptive cells and lysis of mitochondrial contents were observed in the AFB_1_ group. Also, microvilli on the surface of absorptive cells in the jejunum and ileum of the AFB_1_ group were decreased or shedding. Mitochondria play important roles not only in producing ATP, but in controlling apoptosis and contributing to the calcium homeostasis process of cells [37,38]. The involvement of microvilli has been established in various functions such as secretion, mechanotransduction, absorption, and cellular adhesion. Junctional complexes prevent fluid intestinal contents from diffusing into the lamina propria without going through the cells [29]. A terminal web is thought to be responsible for the movement of the microvilli. Damage to the mitochondria and microvilli, along with the disappearance of junctional complexes and the terminal web in some absorptive cells, induced by AFB_1_, showed that AFB_1_ could cause dysfunction of these structures, resulting in functional disorders of absorptive cells in the small intestine. 

To further explore how AFB_1_ impaired the epithelial cells of small intestine, we investigated the number of goblet cells by Alcian Blue/PAS staining. Goblet cells are presumed to protect the mucous membrane in the intestine through the synthesis and secretion of several mediators, such as the mucin MUC2 and the small peptide trefoil factor 3 [39,40]. In this research, AFB_1_ could decrease the numbers of goblet cells, which may contribute to the damage to small intestine epithelia. Furthermore, the mucins production in goblet cells could be up-regulated by TNF-α [41]. Studies have showed that AFB_1_ inhibited the expression of cytokines in the small intestine, including TNF-α [14,42], by which we speculated that the contents of mucins secreted by goblet cells were depressed in the AFB_1_ group. Moreover, goblet cells delivered luminal antigen to CD103^+^ dendric cells in the small intestine [43]. Thus, the decreased goblet cells caused by AFB_1_ may be associated with repressed immunity in the small intestine.

Previous in vivo research has shown that AFB_1_ impaired the adaptive immunity of the small intestine in chickens. Jiang et al. found that AFB_1_ decreased the T cell subset, cytokine expression, IgA^+^ cell numbers, and the expression of immunoglobulin in the small intestine of broilers [14,15]. It is still unknown, however, whether AFB_1_ impaired the innate immunity of the small intestine of chickens in vivo. In this study, we determined the innate immunity of the small intestine of chickens through the expression levels of three toll-like receptors. By qRT-PCR, we found that the expression levels of TLR2-2, TLR4, and TLR7 mRNA were evidently suppressed by AFB_1_ exposure, similarly to zearalenone-induced decrease of TLR-4 in IPEC-1 cells [26] and pig splenocyte [27], and to T2-toxin-induced decrease of TLR-7 in porcine alveolar macrophages [28]. However, mixed aflatoxins B and G could up-regulate TLR2 and TLR4 transcripts in human peripheral blood mononuclear cells [21]. These opposite results may be the consequence of the use of different cell types. Toll-like receptors, expressed on the membranes of immune and non-immune cells, assisted the immune system with the recognition of molecules shared by pathogens, and played vital roles in the innate immune system [44]. TLR2 mediated the host response to Gram-positive bacteria [45], and the functional properties of type 2 TLR2 (TLR2-2) were alike in chickens, humans, and mice [46]. TLR4 could identify lipopolysaccharides (LPS), various viral proteins, polysaccharides, and different kinds of endogenous proteins [47]. TLR7, recognizing single-stranded RNA of viruses such as HCV, played a significant role in the regulation of antiviral immunity [48]. Based on the results of this research, we speculated that AFB_1_ may impair the innate immunity of the small intestine in chickens by depressing TLR2-2, TLR4, and TLR7 mRNA levels. Moreover, the activation of NF-κB in various cell types was triggered by the downstream signaling pathway of TLR2 and TLR4 [49,50]. Following NF-κB activation, various cytokines were released, like IL-6 and TNF-α [51]. Our previous data demonstrated that 0.6 mg/kg AFB_1_ in the broilers’ diet could reduce the expression level of cytokine (like IL-2, IL-4, IL-6, IL-10, IL-17, IFN-γ, and TNF-α) mRNA in the small intestine, implying that the immune function of the intestinal mucosa might be affected [14]. Therefore, the suppressed expression of TLR2 and TLR4 induced by AFB_1_ may contribute to the decreased levels of various cytokines [14,42].

## 4. Conclusions

In conclusion, feed contaminated with 0.6 mg/kg AFB_1_ could induce shedding of intestinal epithelial cells; decrease villus height and area, along with villus:crypt ratio; impair the microvilli and mitochondria of absorptive cells; decrease the goblet cell number; and depress the expression of TLR2-2, TLR4, and TLR7 in the chicken small intestine. These findings indicate that AFB_1_ may decrease the absorptive capacity and partially impair the innate immunity of the small intestine. 

## 5. Materials and Methods

### 5.1. Animals and Groups

Ninety healthy male neonatal Cobb chickens, bought from the Chia Tai Group (Wenjiang, Sichuan, China), were randomized into control and AFB_1_ groups. There were three replicates/group and 15 animals/replicate. Housed in cages with electrically heated units for 21 days, chickens were provided with water as well as the aforementioned diet *ad libitum*. The animal protocols and all procedures of the experiment in this research were carried out according to the laws and guidelines of Animal Care and Use Committee of Sichuan Agricultural University (Approval No. 2012-024). As the chickens were fed with AFB_1_ after hatching, the day of age is the same as the day of the experiment.

### 5.2. Diets 

According to the National Research Council (NRC, 1994) [52] and Chinese Feeding Standard of Chicken (NY/T33-2004), the basal diet was made the control diet. The AFB_1_ (Sigma-Aldrich, St. Louis, MO, USA, A6636) contaminated diet was formulated basically the same as reported earlier [53]. In short, after 27 mg AFB_1_ farinose solid was completely dissolved into 30 mL methanol, the 30-mL mixture was mingled into 45 kg corn-soybean basal diet to formulate the AFB_1_ diet. For the control diet, equivalent methanol was also added into corn-soybean basal diet. Next, the methanol of both food supplies was evaporated at 98 °F (37 °C). Based on analyses by HPLC (Waters, Milford, MA, USA) with fluorescence detection (Waters, Model 2475, Milford, MA, USA), the AFB_1_ concentration was under 0.001 mg/kg in the control group, and 0.601 mg/kg in the AFB_1_ group, respectively.

### 5.3. Histopathological Observation and Microscopic Analyses

The duodenum, jejunum, and ileum from six broilers in each group were collected and fixed in 4% paraformaldehyde on days 7, 14, and 21, and then were dehydrated and embedded in paraffin wax. The sample blocks were sectioned (5 µm) with a microtome (Leica, Wetzlar, Germany, RM2135). The tissue sections were stained with hematoxylin and eosin (H·E), and observed and photographed with a digital camera (Nikon DS-Ri1, Tokyo, Japan).

Five sections of each tissue in a chicken were taken, and five pictures (400×) of each section were taken randomly. The epithelial thickness and villus height, width, and area, as well as crypt depth, were determined by image analysis software (Image-Pro Plus 5.1, Media Cybernetics, Inc., Rockville, MD, USA, 2006). The villus/crypt ratio was calculated by the following formula:

(1) $$\mathrm{Villus}/\mathrm{crypt}\;\mathrm{ratio}\;=\frac{\mathrm{villus}\;\mathrm{height}}{\mathrm{crypt}\;\mathrm{depth}}$$

### 5.4. Transmission Electron Microscope Observation

On day 21, three chickens from each group were humanely killed. At necropsy, the duodenum, jejunum, and ileum were carved into small pieces and immediately put into 2.5% glutaraldehyde for fixation, and in 2% veronal acetate-buffered OsO_4_ for post-fixation. After dehydrating in acetone gradient, the sample tissues were embedded in Epon 812. The sample blocks were sectioned (65–75 nm) in a microtome with a glass knife and put in uncoated copper grids. The tissue sections were stained with uranyl acetate and lead citrate. The ultrastructural architectures of the duodenum, jejunum, and ileum were observed by transmission electron microscope (Hitachi, H-600 transmission, Tokyo, Japan).

### 5.5. Alcian Blue/Periodic Acid-Schiff (PAS) Stain

De-waxed sections were stained in 1% Alcian blue for 5 min, oxidized in 1% periodic acid, immersed in Schiff’s reagent, and mounted and observed by light microscope. With each step, the section was washed in water. The stain showed the goblet cells containing acidic mucins (blue), neutral mucins (magenta), or mixtures of acidic and neutral mucins (purple). Five sections of each tissue in one bird were performed, and five pictures (400×) of each section were taken randomly. The number of goblet cells was calculated on the tip of the villus and the principle for choosing goblet cells was to select the one with more secretion and intact section. All goblet cells in the pictures were counted for further analysis.

### 5.6. qRT-PCR

The small intestines from six chickens in each group on days 7, 14, and 21 were obtained and stored in liquid nitrogen. Then all samples were transferred and stored at −80 °C. Total RNA was extracted using TriPure isolation reagent (Roche Diagnostics GmbH, Mannheim, Germany). The mRNA was reverse-transcribed into cDNA byTranscription First Strand cDNA Synthesis (Roche Diagnostics GmbH). The cDNA was amplified with primers TLR2-2, 4, 7, and β-actin (specified in Table 1) using methods similar to those described by Jiang et al. [14]. Expression of TLR2-2, 4 and 7 transcripts is shown relative to that of β-actin using the 2^−∆∆Ct^ method of Livak and Schmittgen [54].

### 5.7. Statistical Analysis

The results were expressed as the mean ± standard deviation, and the significant difference between the two groups was analyzed by variance analysis, which was performed by the independent sample test of SPSS 17.0 software for Windows. Differences were considered to be statistically significant at *p* < 0.05.

## Reference

## Figures and Tables

**Table 1:** Primers of TLRs and house-keeping genes.

Gene	Primer	Sequences (5′-3′)	Accession Number
TLR2-2	F	CTGGGAAGTGGATTGTGGAC	AB046533.2
	R	CCAGCTCATACTTGCACCAC	
TLR4	F	AGCTACGAGGTTCTGCTCCA	AY064697
	R	TGTCCTGTGCATCTGAAAGC	
TLR7	F	TTATGCCACTCCTCTCTACCG	NM_001011688.2
	R	GCAGCCACCTCTGAAAGATT	
β-actin	F	TGCTGTGTTCCCATCTATCG	L08165
	R	TTGGTGACAATACCGTGTTCA	
